# Vaccinating Front-Line Healthcare Workers: Results of a Pre-Pandemic Cross-Sectional Study from North-Eastern Italy on First Responders

**DOI:** 10.3390/vaccines10091492

**Published:** 2022-09-07

**Authors:** Matteo Riccò, Luigi Vezzosi, Federico Marchesi

**Affiliations:** 1 AUSL–IRCCS di Reggio Emilia, Servizio di Prevenzione e Sicurezza Negli Ambienti di Lavoro (SPSAL), Local Health Unit of Reggio Emilia, Via Amendola n.2, I-42122 Reggio Emilia, Italy; 2 Agenzia di Tutela della Salute (ATS) della Val Padana, Via dei Toscani n.1, I-46100 Mantua, Italy; 3 Department of Medicine and Surgery, University of Parma, Via Gramsci, 14, I-43126 Parma, Italy

**Keywords:** first responders, healthcare workers, knowledge, attitudes, practices, vaccines, immunizations

## Abstract

First responders are front-line healthcare workers who are potentially exposed to different infectious agents. Characterizing their knowledge, attitudes, and practices (KAP) towards immunization, therefore, has the potential to significantly improve occupational health and safety. A cross-sectional study was performed in October 2018 using a sample of 161 first responders from the Parma Province (mean age 45.1 ± 14.1 years; seniority 10.8 ± 8.6 years). The participants were questioned on three recommended vaccinations (i.e., the seasonal influenza, measles, and pertussis vaccines) and on meningococcal vaccines (not officially recommended for first responders). The participant’s knowledge status and risk perception were assessed as percentage values through a specifically designed questionnaire. Adjusted odds ratios (aOR) for factors associated with vaccination status were calculated by means of a binary logistic regression analysis. The internal consistency result, calculated using a general knowledge test, was good (Cronbach’s alpha = 0.894), but the corresponding score was unsatisfying (46.5% ± 32.4), evidencing uncertainties surrounding the recommendations for measles and meningococcal vaccines (39.1% and 34.2% incorrect answers, respectively). While the large majority of respondents were favorable towards the meningococcal (89.4%), measles (87.5%), and pertussis vaccines (83.0%), 55.3% exhibited a favorable attitude toward the seasonal influenza vaccine, the uptake of which in 2018, was reported by 28.0% of respondents, compared to the self-reported lifetime status for meningitis (26.1%), measles (42.2%), and pertussis (34.8%). Not coincidentally, all assessed infections were associated with a low-risk perception score, particularly influenza (33.9% ± 18.4). Interestingly enough, neither knowledge status nor risk perception were associated with vaccination rates. More precisely, the main predictor for being vaccinated against seasonal influenza in 2018 was a seniority of ≥10 years (aOR 3.26, 95% confidence interval [95% CI] 1.35–7.91), while both pertussis and measles were positively associated with higher educational achievement (aOR 3.27, 95%CI 1.29–8.30; and aOR 2.69, 95%CI 1.09–6.65, respectively). The reasons for vaccination gaps among the sampled first responders, apparently, did not find their roots in inappropriate knowledge status and risk perception alone. However, the very low rates of sampled immunization lead us to recommend stronger and more appropriate information campaigns.

## 1. Introduction

First responders (i.e., paramedics, emergency medical technicians, ambulance personnel, firefighters) are front-line healthcare workers (HCWs), who are occupationally exposed to different infectious agents [1,2,3] through frequent and close contact with high-risk patients in a work environment that is often uncomfortable (as it encompasses ambulances, private houses, outdoor settings, etc.), and deprived of those preventive assets that are otherwise available in common healthcare settings [4,5]. Because of their potential interaction with high-risk groups [2,6,7,8,9], they represent a possible source of infection for susceptible patients or colleagues [10,11,12], and are therefore targeted by competent public health authorities through specific vaccination policies [10,13,14,15,16,17,18,19]. As “essential workers”, first responders perform work that involves the safety of human life or the protection of property. Immunization policies aim to reduce their risk of requiring sick leave, particularly during epidemic/pandemic events (e.g., seasonal influenza) [10,17,18,19]. Even though interest in first responders and their vaccinations status has usually focused on bloodborne pathogens, such as HBV, because of the high risk of blood exposure while handling patients and equipment [5], the ongoing SARS-CoV-2 pandemic has stressed how an appropriate and up-to-date vaccination status against respiratory pathogens is also necessary in order to guarantee the health and safety of both patients and first responders HCWs [19,20,21,22].

In Italy, the Italian National Immunization Prevention Plan (in Italian: “Piano Nazionale della Prevenzione Vaccinale”, PNPV) 2017–2019, in line with the available evidence, specifically recommends HCWs to undergo vaccination against influenza, pertussis, measles, parotitis, rubella, varicella, and Hepatitis B in the belief that their vaccination will reduce absenteeism and the risk of work-related infections [11,12,23,24]. Nonetheless, in 2017, a position paper, the so-called “Pisa card”, was signed by the Italian Multidisciplinary Society for Infection Prevention in Health Organizations, the Italian Society of Occupational Health, and the Italian Society of Hygiene, in order to actively promote (among HCWs) those vaccinations which are not covered by a specific mandate [25]. Even more tightened recommendations have been issued by competent health authorities in some regions, such as Emilia Romagna, Apulia, and Marche [26]. These resolutions are based upon the national Law on Workers’ Health and Safety in the Workplace (Legislative Decree no. 81/2008) and define “fitness to work” as strictly depending on the HCWs’ vaccination status. However, significant uncertainties still remain regarding the requirements for first responders operating as volunteers. This is a significant issue, as in Italy, volunteer rescuers who usually lack any formal medical education respond to most pre-hospital emergency calls [27].

The knowledge, attitudes, and practices (collectively, KAPs) of first responders towards immunization, therefore, have the potential to significantly affect public health and occupational health [10,13,14,15,16,17,18,19,20,21]. Unfortunately, the abovementioned issues have been scarcely investigated [4,28,29,30]. Therefore, in this questionnaire-based cross-sectional study conducted shortly before the occurrence of the SARS-CoV-2 pandemic, we assessed the knowledge of a sample of first responders regarding VPDs and their official recommendations (i.e., knowledge of PNPV 2017–2019 recommendations), as well as their attitudes and personal beliefs, and whether knowledge, attitudes, and personal beliefs may be predictive of their vaccination status. More specifically, we focused on four immunizations towards non-bloodborne pathogens, i.e., the seasonal influenza vaccine (SIV), measles vaccine (MeV), pertussis vaccine (Pa) and meningitis vaccine (MEN). While SIV, MeV, and Pa have been specifically targeted by guidelines and official recommendations for HCWs. At the moment, the MEN (B/C/ACWY) is not specifically recommended for HCWs [11,12,23,24,26]. However, the MEN was deliberately included in the analyses because of the potentially dire consequence of an occupational contagion in ambulance workers and the significant risk perception of HCWs [2].

## 2. Materials and Methods

### 2.1. Study Design

In this cross-sectional questionnaire study (See STROBE checklist as Supplementary Material File S1), first responders operating in the Province of Parma, Italy, were asked about their KAPs toward four VPDs, for which airborne transmission represents a potential threat for ambulance workers, i.e., pertussis, meningitides, influenza, and measles [31,32].

### 2.2. Study Population

**Figure 1:**
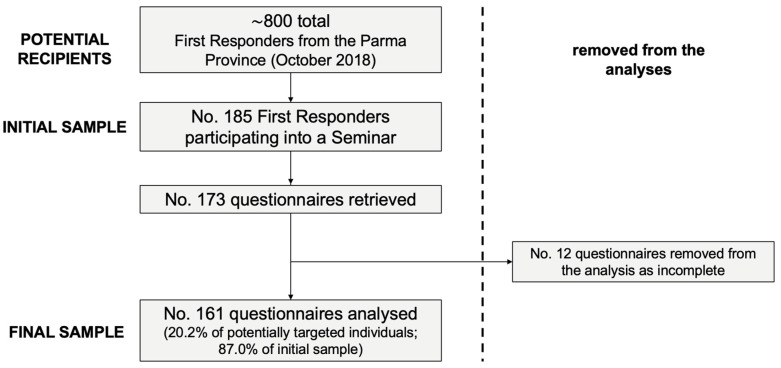
Flow chart of sampled participants.

A seminar on the immunization policies put in place via the PNPV 2017–2019 took place in October 2018. Before the start of the seminar, all participating first responders (No. 185) were asked whether they would agree to participating in a survey about knowledge and attitudes towards vaccinations. The collected sample eventually included 161 professionals, representing 20.2% of all first responders operating in the Province of Parma during December 2018 and around 87.0% of the professionals participating in the educative intervention (Figure 1).

### 2.3. Ethical Considerations

Before they gave their consent, participants were briefed and told that all information would be gathered anonymously, handled confidentially according to the guidelines of the Declaration of Helsinki, and safely stored for the time required by the present analysis (translation of the informed consent is available via Supplementary Material File S2). Moreover, all retrieved personal data (e.g., age, gender, and educational level) were strictly finalized for the present analyses, and individual participants cannot be identified based on the presented material. Participation was voluntary, and the questionnaire was collected by hand at the end of the meeting only from those subjects who expressed formal consent for study participation, causing no plausible harm or stigma to participating and non-participating individuals. As the study had an observational design, lacking in clinical data about the respondents, it did not configure itself as a clinical trial, being characterized rather as an “opinion survey”. Its preliminary evaluation by an ethical committee was therefore not required, according to Italian law (Gazzetta Ufficiale no. 76, dated 31 March 2008).

### 2.4. Questionnaire

Our inquiry was performed through an adapted and translated version of the utility previously developed by Zingg and Siegrist [31,32]. An Italian translation was previously employed in several KAP studies [23,33,34,35]. Briefly, the questionnaire comprised some general demographic information (i.e., age, sex, country of origin, seniority as first responder, and educational achievements) and contained 22 items divided into four areas of inquiry, as follows:

#### 2.4.1. General Knowledge

The present knowledge test contains true/false statements, such as “vaccinations increase the occurrence of allergies” (false), covering some typical misconceptions on vaccination [32], and the sum of all incorrect answers was interpreted as the degree of misconceptions held by the individual participant [23,32,33,34,36]. In similarly designed KAP study, this test successfully predicted influenza risk perception and vaccination intention for various target populations [23,32,33,34,36]. Briefly, a total of 13 statements were presented, and general knowledge was then calculated as the sum of correctly and incorrectly marked recommendations. When the participant answered correctly, +1 was added to a sum score, whereas a wrong indication or a missing/“don’t know” answer added 0 to the sum score. The potential score ranged, therefore, from 0 to 13.

#### 2.4.2. Knowledge of Official Vaccination Recommendations

A total of 16 vaccine-preventable diseases were initially presented (i.e., diphtheria, tetanus, pertussis, poliomyelitis, viral hepatitis A, viral hepatitis B, influenza, pneumococcus, *Haemophilus influenzae*, measles, rubella, parotitis, varicella, meningococcus, human papillomavirus (HPV), and tuberculosis). For each disease, participants indicated whether they thought that PNPV 2017–2019 recommends vaccination for HCWs (possible answers: “yes”, “no”, “don’t know”). Knowledge regarding the official vaccination recommendations was calculated as the sum of correctly and incorrectly marked recommendations; when the participant correctly indicated a vaccination as recommended or not recommended by the Italian National Vaccine Prevention Plan, +1 was added to a sum score, whereas a wrong indication or a “don’t know” answer added 0 to the sum score. As the ongoing recommendations for HCWs include seven immunizations (i.e., pertussis, viral hepatitis B, influenza, measles, rubella, parotitis, varicella), the potential score ranged from 0 to 7.

#### 2.4.3. Risk Perception

Perceived risk has been otherwise interpreted as the product of the perceived probability of an event and its expected consequences [31,34,35]. Therefore, we asked the first responders about their perceived probability (P) and severity (S) of natural infection and vaccine-related adverse effects through a fully labeled five-point scale: “very low” (score = 1), “low” (score = 2), “moderate” (score = 3), “high” (score = 4), and “very high” (score = 5). Each one of the four assessed VPDs was separately assessed. A distinctive, cumulative risk perception score (RPS) was then calculated as the mathematical product of P × S for each one of the four vaccinations and for the four VPDs (potential range 1 to 25).

#### 2.4.4. Attitudes

First responders rated their general attitude towards the acceptance of the four VPD vaccinations through a five-point Likert scale (the values were “absolutely against”, “somewhat against”, “neutral”, “somewhat favorable” and “absolutely favorable”). Eventually, the reported attitudes were dichotomized into somehow favorable (values: “somewhat favorable” and “absolutely favorable”) vs. somehow against (values: “neutral” to “absolutely favorable”). Participants who were somehow favorable towards SIV were then asked to report which factors are perceived as promoting seasonal immunization, while participants who reported a somehow unfavorable attitude were similarly requested to identify which barriers they perceived as being more significant.

#### 2.4.5. Practices

As some further specific recommendations and some mandatory immunizations have been put in place for HCWs in the Emilia Romagna Region since 2017, participants were initially requested to report whether they worked in healthcare settings or not. Then, they were asked about their immunization status. A completed and up-to-date vaccination was defined as follows: two shots for measles, one booster shot against pertussis within the last 10 years, and one shot for SIV for either the influenza season 2017 and/or 2018 (separately assessed). Regarding meningococcus, as several serotype-specific vaccines are available, any previously reported vaccination was considered appropriate. 

### 2.5. Data Analysis

There were two independent researchers: one of whom read the responses from each questionnaire, while the other researcher reviewed the entered data and ensured their accuracy. Doubtful cases (i.e., heterogeneous interpretation by researchers involved in data entry) and unclear responses were reviewed by the primary investigator (MR) in order to determine which answer was to be assumed as “*correct*”. Questionnaires lacking basic information about the interviewee were excluded from the study. A preventive reliability test was performed on the general knowledge section through the determination of Cronbach’s alpha. We calculated the described indices for general knowledge (i.e., General Knowledge Score, GKS), knowledge about PNPV (PNPV-KS), and risk perception, as previously described. In order to more easily compare the scales, all cumulative scores were initially normalized to percentage values (min: 0.0, max: 100) and then dichotomized into a high vs. low score by the median value.

Continuous variables were initially expressed as mean ± standard deviation (SD). Categorical variables were reported as percentage values. Univariate association of demographic factors, such as age (<50 years-old vs. ≥50 years-old), sex, migration background (Italian born vs. foreign born people), seniority as a first responder (<10 years vs. ≥10 years), educational achievements (<University vs. ≥University level) and individual factors (i.e., occupational background in healthcare settings vs. other background, high vs. low scores for GKS, PNPV-KS, RPS, and favorable vs. not favorable attitude towards the specific vaccine) and the self-reported immunization status (i.e., yes vs. no/don’t remember) for the assessed VPDs (i.e., SIV 2018, measles, pertussis, and meningococcus) were initially evaluated through Chi-squared testing (with continuity correction), while the adjusted odds ratios (aOR) with the respective 95% CI were calculated through binary logistic regression analysis. Regression analysis included, as covariates, demographic factors irrespective of their actual association with vaccination status in univariate analysis, while only individual factors significantly associated with a positive vaccination status were eventually included. The significance level was <0.05 for all calculations. All analyses were performed by means of SPSS 24 (IBM Corp. Armonk, NY, USA).

## 3. Results

### 3.1. Characteristics of the Sample

As shown in Table 1, the final sample included a total of 161 respondents. Of these, 64.0% were males; 42.9% were aged 50 years or older (mean age 45.1 ± 14.1 years), while 47.8% reported seniority as a first responder of 10 years or more (mean 10.8 ± 8.6 years), and 5.0% had a migration background. The majority of the participants reported a high (secondary school/high school: 65.2%) or even very high (university level: 26.7%) education achievement. Focusing on their occupational background, 11.8% were employed in healthcare settings.

### 3.2. Knowledge Status

**Figure 2:**
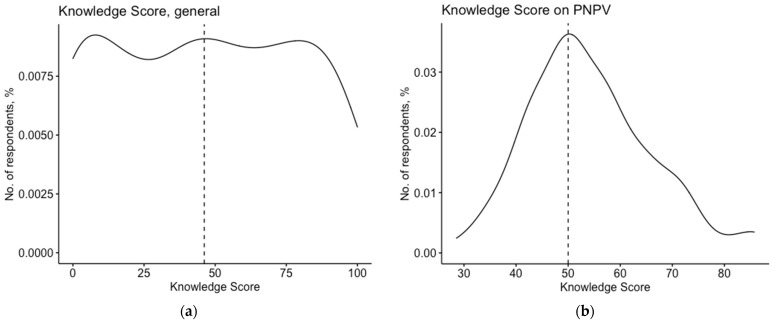
Density plots for knowledge scores, calculated for general knowledge on vaccinations (average: 46.5% ± 32.4, range 0–100; median 46.2%) (**a**) and on the understanding of the National Immunization Prevention Plan 2017–2019 (PNPV) (average: 54.1% ± 11.8, range 28.6–85.7; median 50.0%) (**b**). Dotted lines report the respective median values.

After percentage normalization, the mean GKS was 46.5% ± 32.4, with a substantially skewed distribution (D’Agostino–Peason’s *p* value = 0.015). Cronbach’s alpha for the knowledge test was equal to 0.894 (Figure 2a).

Focusing on the single statements (Table 2), the majority of the respondents were aware that vaccines allowed for smallpox eradication (67.7%), that their efficacy has been extensively proven (64.0%), and that vaccines are instrumental in treating infectious diseases, as appropriate antibiotic treatment is not always possible (59.6%). Still, the majority of the participants exhibited significant uncertainties and a large share of false beliefs on the remaining statements, as none of these statements were correctly characterized by 50% or more of the respondents. More specifically, only a third of the participants were aware that vaccines do not increase the occurrence of autoimmune diseases (32.3%), that the number of pediatric vaccines does not threaten to overwhelm the immune system (37.9%), and that the additives used in the vaccines are not dangerous to humans (37.9%). 

When dealing with the perceived status of the presented immunizations, a cumulative PNPV-KS of 54.1% ± 11.8 was identified (Figure 2b). The correspondent distribution was substantially skewed (D’Agostino–Pearson’s test *p* value < 0.001). The large majority of the participants were aware that official recommendations for HBV vaccine (91.9%) and SIV (88.2%) do exist for HCWs, while more significant uncertainties were reported regarding the vaccinations, including the MPR-V formulate (Measles, 60.9%; Parotitis, 42.9%; Rubella and Varicella, both immunizations 51.6%), and more specifically for pertussis (39.8%). Interestingly enough, the majority of the respondents incorrectly reported the official recommendations for meningococcal vaccines (65.8%).

### 3.3. Attitudes

As shown in Table 1, most of the respondents were either favorable or highly favorable towards the MEN vaccine (89.4%), followed by the MeV (87.5%) and Pa (83.0%) vaccines, while only 55.3% of the participants exhibited this attitude towards SIV. Among the subjects somehow favorable to SIV, the main promoting factors were identified as avoiding the spread of seasonal influenza (75.3%), protecting subjects who cannot be vaccinated (74.2%), followed by avoiding complications (67.4%), and being totally immune (46.1%). While 14.6% reported specific recommendations for SIV, only 11.2% of the participants recommended a healthcare provider, either a general practitioner (5.6%) or an occupational physician (5.6%). Focusing on the perceived barriers, the most frequently reported barrier was the lack of trust in SIV (44.4%), followed by the preference for other preventive measures (37.5%), the perception of SIV as something “unnecessary” (i.e., the respondent identified himself/herself as “otherwise immunized”) (15.3%), and the fear of side effects (13.9%). Moreover, only a few respondents perceived SIV as an ancillary measure when compared to the lifestyle of preventing influenza (2.8%) or totally useless (2.8%), while only one respondent reported fear of injection as a significant barrier.

**Figure 3:**
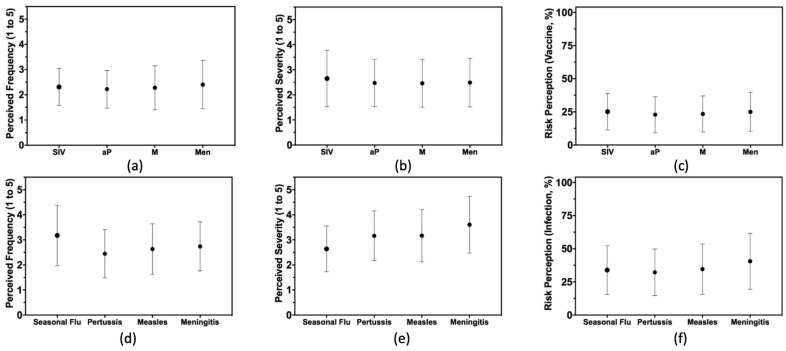
Risk perception component for 161 first responders from the Province of Parma (2018) participating in our survey. Participants were questioned on four vaccine-preventable disorders (i.e., seasonal influenza, pertussis, measles, meningococcal meningitis), focusing on the perceived frequency and severity of both vaccine side effects and natural infection. Respective risk perception scores for natural infection and vaccine side effects were calculated as the product of the perceived frequency and severity of the events (Note: SIV = Seasonal Influenza Vaccine, aP = Pertussis vaccine, M = measles vaccine, Men = meningitis vaccine; Subfigures: (**a**) = perceived frequency of vaccine side effects; (**b**) = perceived severity of vaccine side effects; (**c**) = risk perception score for vaccinations; (**d**) = perceived frequency of natural infections; (**e**) = perceived severity of natural infections; (**f**) = risk perception score of natural infections).

**Figure A1:**
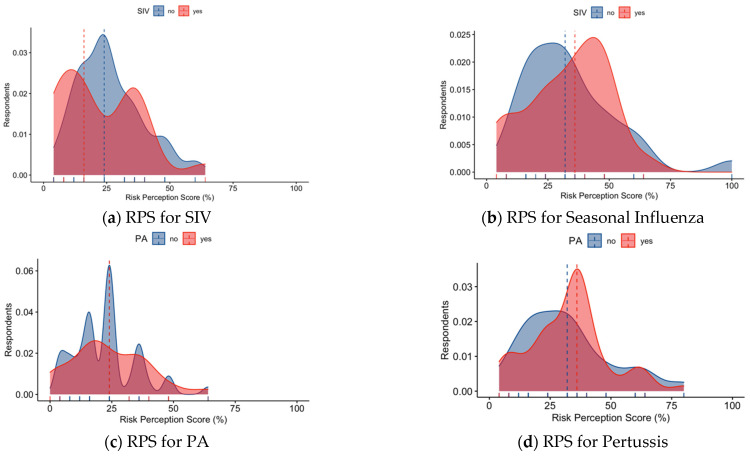

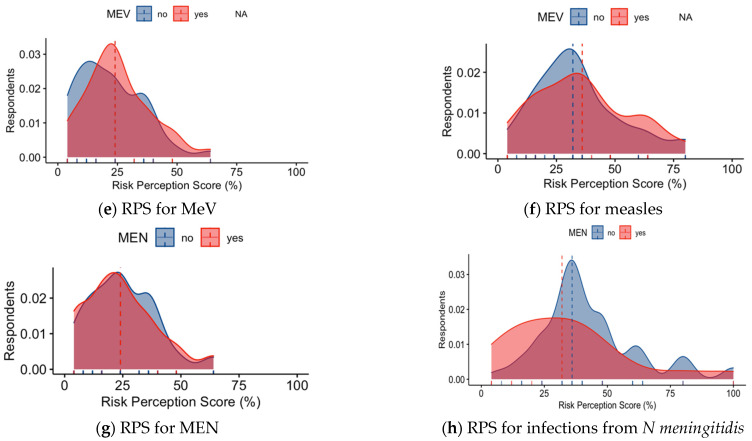
Risk perception score for: (**a**) seasonal influenza vaccine (SIV), (**c**) pertussis vaccine (PA), (**e**) measles vaccine (MeV), (**g**) meningitis from *N meningitidis* (MEN), and corresponding natural infections: seasonal influenza (**b**), pertussis (**d**), measles (**f**), infections from *N meningitidis* (**h**), all of them broken down by having or having not been vaccinated (SIV: 2018; PA, MeV, MEN = lifetime).

When dealing with the RPS associated with natural infections (see Figure 3), participants scored a higher risk perception for natural infection for *Neisseria meningitidis* (40.5% ± 21.0), followed by measles (34.6% ± 18.9), the influenza virus (33.9% ± 18.4), and eventually *Bordetella pertussis* (32.2% ± 17.5). On the contrary, higher concerns were formulated against SIV (25.1% ± 13.8), followed by MEN (25.0% ± 14.6), MeV (23.4% ± 21.0) and Pa (22.9% ± 21.0) (see Annex Figure A1).

### 3.4. Practices

Overall, the single most frequently recalled adult vaccination was for measles (42.2%), followed by pertussis immunization (34.8%), while SIV was recalled by 28.0% and 26.1% of the respondents for the 2017 and 2018 influenza season, respectively, with any meningococcal vaccination recalled by 26.1% of the participants.

### 3.5. Univariate Analysis

GKS and Knowledge of PNPV were positively correlated (Spearman’s rho = 0.179; *p* = 0.023). In turn, as shown in Table 3, GKS was positively correlated with RPS for N meningitidis infection (rho = 0.161; *p* = 0.042), i.e., a better knowledge of vaccine-related issues was associated with a higher risk perception for meningococcal diseases. On the contrary, GKS was negatively associated with the risk perception of all of the assessed immunizations. In other words, an improved knowledge status was associated with lower concerns regarding the risks of immunizations.

In the univariate analysis (Table 4), the assessed vaccinations exhibited proper, distinctive patterns. More precisely, having been vaccinated in 2018 against seasonal influenza was significantly associated with being male (77.8% vs. 58.6%, *p* = 0.037), of a seniority of ≥10 years (68.9% vs. 39.7%, *p* = 0.002), reporting an occupational background of healthcare settings (22.2% vs. 7.8%, *p* = 0.023), scoring higher concerns towards natural infection (60.0% vs. 26.2%, *p* = 0.010), and having a somehow favorable attitude towards SIV (80.0% vs. 45.7%, *p* < 0.001). Focusing on the pertussis and measles vaccines, on the one hand, a previous immunization was more scarcely reported in subjects in an older age group (21.4% vs. 54.3%, *p* < 0.001, and 19.1% vs. 60.9%, *p* < 0.001, for measles and pertussis, respectively), and having greater seniority (28.6% vs. 58.1%, *p* < 0.001; 30.9% vs. 60.9%, *p* < 0.001). On the other hand, higher rates were reported from subjects having a migration background (12.5% vs. 1.0%, *p =* 0.005; 10.3% vs. 1.1%, *p* = 0.023), reporting higher educational achievement (37.5% vs. 21.0%; 36.8% vs. 19.6%; *p* = 0.025), and scoring a higher risk perception for natural infection (55.4% vs. 36.2% for pertussis; 54.4% vs. 38.0%, *p* = 0.038 for measles).

Furthermore, higher vaccination rates were identified, only for measles, in subjects from a healthcare background. Interestingly enough, while a somehow lower vaccination rate was identified in subjects with a higher education achievement level (16.7% vs. 30.3%, *p* = 0.132), only age was significantly and negatively associated with a previous meningococcal vaccination (26.2% vs. 48.7%, *p* = 0.018).

### 3.6. Multivariable Analysis

Overall (Table 5), SIV 2018 was positively associated with higher seniority (aOR 3.262, 95% CI 1.346 to 7.905), higher risk perception for natural infection (aOR 3.374, 95% CI 1.367 to 8.332), and a more favorable attitude (aOR 8.404, 95% CI 3.070 to 23.009). Both pertussis and measles immunizations were positively associated with higher educational achievements (aOR 3.274, 95% CI 1.291 to 8.304, and aOR 2.693, 95% CI 1.090 to 6.651, respectively) and negatively with higher seniority (aOR 0.198, 95% CI 0.080 to 0.489 for pertussis, aOR 0.197, 95% CI 0.085 to 0.445 for measles), while pertussis vaccination was positively associated with being born outside Italy (aOR 15.330, 95% CI 1.418 to 165.737), and reporting a healthcare settings background (aOR 10.898, 95% CI 2.638 to 45.017). In turn, being reportedly vaccinated against measles and Neisseria meningitidis was negatively associated with being aged 50 years or older (aOR 0.114, 95% CI 0.047 to 0.278, and aOR 0.316, 95% CI 0.142 to 0.704, respectively), while the referral of any meningococcal vaccination also negatively associated a university-level education background (aOR 0.376, 95% CI 0.146 to 0.964).

## 4. Discussion

Guaranteeing a high level of immunity against airborne VPDs is considered particularly important for healthcare professionals [10,11,12,37], and the recent SARS-CoV-2 pandemic has stressed how vulnerable HCWs may be to these pathogens if they are requested to face them without appropriate personal protective equipment and/or vaccine prophylaxis, particularly when their risk perception of the potential threat is disproportionately low [38,39,40]. In the present study, the majority of the respondents were favorable or even highly favorable toward the reported immunizations, particularly toward meningococcal disorders (89.4%) and the measles virus (87.5%). The reported vaccination rates, if accurate, were largely unsatisfactory. In fact, not only was a previous and effective uptake of MeV reported by less than half of the respondents (42.2%), but around one-third of them had previously been vaccinated against pertussis (34.8%), with even lower estimates for the seasonal influenza virus (28.0% for SIV 2018, 26.1% for SIV 2017), and MEN (26.1%). In order to fully appreciate these vaccination rates as being unsatisfactory, the reader should keep in mind that in Italy, SIV, MeV, and Pa vaccination are free of charge for adults in high-risk groups as first responders, while MEN vaccines need to be paid for by the recipient [15,41]. On the other hand, it should be stressed that the present study was performed well before the emergence of SARS-CoV-2 (i.e., October 2018), when inappropriate vaccination rates and a struggling acceptance of SARS-CoV-2 vaccines led health authorities to implement mandatory SARS-CoV-2 vaccination policies [42,43,44,45]. 

Even though the background to this study has, therefore, radically changed since the delivery and collection of the questionnaires, our results might contribute to improving our understanding of the root causes that have initially harmed the kick-off of the COVID-19 vaccination campaign and to better circumscribe those critical issues to be targeted in order to improve vaccination rates in first-line HCWs.

In fact, our results are also quite consistent with previous reports from Italian healthcare settings [6,12,13,46,47], the background of which has been extensively but also inconclusively assessed throughout the last decade [48,49,50]. Usual explanations for the relatively scarce acceptance of vaccinations in HCWs, particularly among Italian HCWs, are the shared lack of knowledge, the scarce risk perception, and often vague individual, “emotional” factors [11,37,43,49,51]. For example, there is a consolidation of evidence that vaccination acceptance is directly influenced by trust in the vaccine efficacy, safety, and potential benefits [4]. However, it is important to stress that the drivers for vaccine acceptance and/or hesitancy may be quite heterogenous, not only in various targeted populations but also in-between the assessed immunizations [11,24,26,49,52,53,54,55,56,57,58,59,60]. Not coincidentally, a negative correlation was found between GKS and risk perception for individual vaccinations, but in turn, a better knowledge score was not substantially associated with the assessed vaccinations. In other words, personal characteristics (e.g., education level, source of information, and demographics) that motivate the acceptance of a certain vaccine may, in fact, lead to the refusal of another one, eventually explaining certain inconsistencies among various studies which target the very same study population. 

For one, belonging to an older age group was characterized as a negative effector for Pa, MeV, and MEN immunizations; when dealing with Pa and MeV, a possible explanation may be found in the usual underestimation of severity and frequency of pertussis and measles in the adult population, particularly in Italy [12,48,61,62,63,64,65,66,67,68,69], as indirectly suggested by the higher acceptance of the Pa vaccination among those respondents with a migration background. When dealing with the low acceptance of MEN immunization, it is important to stress that invasive *Neisseria* infections in adults are relatively rare in Italian settings [2,70,71,72], particularly among healthcare workers [2]. Even though *N meningitidis* infections may lead to dire clinical consequences, it is usually perceived as a pathogen in children and adolescents, as shown by a recent cross-sectional study on a sample of Italian parents [70]. Not coincidentally, within a percentage scale, even a potentially lethal pathogen like *N meningitidis* was characterized by a surprisingly low-risk perception (40.5% ± 21.0), which was not significantly greater than that acknowledged for measles (34.6% ± 18.9), the influenza virus (33.9% ± 18.4), and *Bordetella pertussis* (32.2% ± 17.5). When dealing with the risk perception of the reported vaccines, overall scores resulted in relatively low estimates for all of the assessed vaccinations (i.e., SIV 25.1% ± 13.8; MEN 25.0% ± 14.6; measles 23.4% ± 21.0; and pertussis 22.9% ± 21.0), but only 55% of participants were favorable to SIV, and nearly half of individuals not favorable to receiving SIV reported some degree of mistrust towards this particular immunization.

Second, a somehow contradictory effect of education was found when dealing with the acceptance of MeV and Pa compared to MEN. While a higher education level was associated with better acceptance of the former immunizations, it resulted in a more negative attitude towards MEN. On the one hand, a better education, particularly when achieved in settings other than healthcare, does not univocally guarantee better acceptance of medical interventions, including vaccines [4,73,74,75,76], as recently shown by studies on KAP studies on mRNA vaccines [55,77,78,79], and HCWs are not spared from this [55,57,80,81]. On the other hand, we can speculate that individuals with higher education attainment may have been made more familiar with the recent requirements issued by Italian governments and health authorities on these specific immunizations [15,41,43]. In this regard, it should be stressed that MEN immunizations were generally not considered in Italian vaccination guidelines in settings other than the pediatric general population [15,41,70]. Combined with low incidence rates, this may have led to a general underappreciation of the potential occupational significance of this immunization [2,70,71,72].

Eventually, higher seniority was positively associated with better acceptance of SIV, being, conversely, a negative effector for MeV and Pa. While a lower acceptance for MeV and Pa may be explained through the scarce appreciation of these disorders, particularly among adults [12,48,62,82,83,84,85,86], several explanations may be suggested for the role of seniority in improving SIV rates. On the one hand, SIV is usually associated with lower rates of sick leave among recipients [46,49,52,87]. Other than solidaristic factors, such as avoiding the spread of the infection among familiars and patients, older first responders are more likely to appreciate the practical impact of higher vaccination rates in terms of reduced absenteeism during the flu season [3,13,88,89,90,91].

On the other hand, as stressed by multivariable analysis, factors underlying the acceptance of SIV are particularly complex, with deep roots in an individual’s understanding of this disorder, as explained by means of the health belief model [92,93]. The rationale of the health belief model is that beliefs about the susceptibility to a health threat correspond with perceptions about the severity of that threat, and the perceived benefits and barriers associated with a particular protective action will determine whether or not an individual will adopt that action [94,95]. As influenza is usually acknowledged as a relatively indolent disorder, the corresponding vaccine has been, in turn, often discredited by the media [13,14,47,96,97,98,99]. HCWs may doubt their individual benefit from being vaccinated. Therefore, increased acceptance of the vaccine is expected in individuals characterized by higher risk perception, regardless of cause, as previously stated by several studies on HCWs [46,52,87,100]. In fact, also in our report higher risk perception was characterized as the main positive effectors for receiving SIV [13,14,47,96,97,98,99]. Even among individuals somehow favorable to SIV, the aim of avoiding the natural infection was not reported as a main driver, being preceded by solidaristic (i.e., avoiding the spread of seasonal influenza, 75.3%, and protecting those cannot be vaccinated, 74.2%).

In other words, not only is our study consistent with previous claims on the complex mechanisms leading to the acceptance and/or the refusal of recommended health interventions [54,93,101,102,103], but it also suggests that first responders are not spared by uncertainties and false beliefs about vaccines and immunizations, and that such knowledge gaps may model their attitudes in an effective but undesired way. In our study, knowledge status was largely unsatisfactory, particularly for general knowledge of vaccine-related issues (46.5% ± 32.4). First responders were extensively affected by knowledge gaps and false beliefs, as the large majority of respondents failed to dismiss the alleged side effects of vaccinations in younger age groups. More specifically, the effect of MeV on autism and neurological disorders. However, official recommendations by the HCWs were also characterized by some uncertainty (54.1% ± 11.8), particularly for the immunization included in the MPR-V vaccine, while the mandatory status for the MEN vaccine was mistakenly reported. It must be acknowledged that HCWs are often affected by vaccine hesitancy because of diffuse false beliefs or even having trust in “fake news”, not differentially to those in the general population [10,19,23,24,26,33,43,49,53,81,104,105,106,107]. In fact, as first responders are HCWs who often lack formal education and some professional expertise, they may be particularly vulnerable to these potentially detrimental factors [10,17,18,19], as suggested by our results.

*Limitations*. Despite its potential significance, even in the daily practice of public health professionals (e.g., by designing specific vaccination programs and improving specific vaccination policies), our study is affected by several shortcomings which must be acknowledged. 

For one, we must still acknowledge that our sample was relatively small and included only first responders from a very delimited geographic area (i.e., the Parma Province in north-eastern Italy). As a consequence, the results drawn from the statistical analysis should be carefully examined. More precisely, assuming as an a priori hypothesis that half of the participants exhibited a favorable attitude towards the reported vaccinations, for a type I error of 0.05, a minimum sample size equal to 1.96^2^ × 0.5 × (1 − 0.5)/0.05^2^ = 3.8416 × 0.5 × 0.5/0.0025 = 384 may be calculated, as compared to the 161 first responders actually participating in our survey [108]. Moreover, the study sample collected occurred out of convenience (first responders participating in a formative event), so its generalizability should be even more carefully assessed. Even though we were able to recruit around one-fifth of all potentially targeted first responders from the Province of Parma, the generalizability of the reported results for this area may also be questioned. Still, it should be stressed that the province of Parma was directly involved in the first wave of the SARS-CoV-2 pandemic [109,110,111]. For instance, on 30 April 2020, the Emilia Romagna Region had a cumulative case rate of 570 cases per 100,000, compared to the national estimate of 341, with most of these reported from within the provinces of Piacenza, Parma, and Reggio Emilia. Between March and May 2020, HCWs, particularly first responders from these provinces, faced an unprecedented surge in their daily requirements within a multidimensional framework that not only included the on-site intervention of incident cases but also the reallocation of patients from the most affected areas to other hospitals within both regional and national networks. Ultimately, these requirements have taken a substantial toll in terms of work-related infections and deaths [39,43,111,112,113], as certified by the official statistics from the National Institute for Insurance Against Accidents at Work (INAIL) [114]. Taking 7.4% of the total Italian population, Emilia Romagna reported 10.3% of all work-related cases of COVID-19, as well as 21 out of 252 incident deaths (8.3%). Parma, accounting for 10.1% of the regional population, reported around 12.9% of all cases, and around a fifth of these (19.8%) occurred in out-of-hospital frontline HCWs [114,115].

As a consequence, despite the limited significance of our results in explaining the more complex phenomenon of vaccine hesitancy among HCWs, our study may share a sort of “historical” picture of the acceptance of vaccination policies among a front-line group of Italian first responders, shortly before their involvement in an unprecedented event, as represented by the COVID-19 pandemic. Nonetheless, the high degree of false beliefs about the assessed disorders and vaccines, particularly about respiratory disorders, hints that (particularly in the early stages of the pandemic) most first responders may have not correctly faced this new pathogen, ultimately exposing themselves to a high risk of SARS-CoV-2 infection.

Second, we must acknowledge the fact that “not participating” could be understood as a negative attitude or a lack of knowledge about the targeted topic [116]. As a large share of the first responders attending the educative intervention did participate in the survey (i.e., 87.0%), potential self-selection within the respondents for participation in the parent seminar cannot be ruled out. Moreover, seeing as the questionnaires were collected at the end of the educative intervention, we cannot rule out that the knowledge status of the participants may have been somehow improved and that, similarly, their real-world acceptance of the assessed vaccinations may be even lower than that reported in the present study. In this regard, it is particularly important to stress that, by its design, this survey has measured the knowledge of the participants of the assessed vaccinations at the time of the survey, whereas, by asking first responders about their risk perception, we have reasonably retrieved a proxy of their attitudes prior to this survey and the educative intervention seminar, leading to a potential logical problem. Similarly, having been (or not having been) previously vaccinated against the selected VPD could present past information, not reflecting the actual attitudes of the participants at the time of this study. A possible proof may be identified in the negative association between knowledge status and concerns for the individual vaccinations, while no clear correlation was found between risk perception of the vaccinations and vaccination status. This specific bias may be particularly difficult to address because of the small size of the sample. However, risk perception is a far more complex domain than that of the measurement of the basic knowledge of a specific health topic, and a single intervention may hardly have led to a radical change in attitudes towards a specific immunization [95,117]. In fact, there is some evidence that vaccine hesitancy, despite its well acknowledged continuous range from total acceptance to the total refusal of vaccinations [118,119], may follow discrete steps, as summarized by the transtheroretical model of health behavior change [117,120,121], limitedly impairing the internal consistency and the underlying rationale of similarly designed surveys [31,32].

Third, we cannot rule out the fact that some of the items we assessed through the knowledge test may have been affected by a significant social desirability bias, as was previously suggested by other studies with similarly designed questionnaires [31,32,43,106,107,122]. A substantial share of the participants may have, therefore, reported those that they felt as “common sense” answers as perceived, more “appropriate answers”, with the eventual overstating of the participants exhibiting an effective understanding of reported immunizations and related associated issues. The social desirability bias may be quite difficult to counter, and it is quite reasonable that the implementation of a vaccination mandate in Italian healthcare settings may amplify the magnitude of this factor in follow-up studies.

Last but not least, the very same settings of our inquiry may represent both a substantial limit and a point of strength for the present study, which was clearly unexpected at the time of its inception. As this study was designed and performed before the onset of the SARS-CoV-2 pandemic, we cannot rule out that both the overall understanding and acceptance of the vaccinations may have been radically changed by the daily experience of the first responders, with follow-up studies having only limited comparability with our results. Because of the substantial share of HCWs still exhibiting vaccine hesitancy well after the SARS-CoV-2 pandemic, an updated inquiry of this specific subgroup of HCWs may be particularly useful in order to specifically design appropriate informative and formative interventions.

## 5. Conclusions

In conclusion, despite some significant limits, our study suggests that shortly before the occurrence of the SARS-CoV-2 pandemic, our sampled first responder HCWs were affected by significant knowledge gaps and false beliefs about several vaccine-preventable diseases, which eventually led to an improper acceptance of the assessed vaccinations (i.e., MeV, MEN, SIV, and PA). In order to cope with the requirements of this specific occupational group and improve the overall safety status of their patients, a more tailored formation should be guaranteed. Despite the limits of the present study, particularly the small sample size, our methodology could be implemented in future studies that monitor the knowledge status of HCWs on vaccine-preventable disorders.

## Figures and Tables

**Table 1:** General characteristics of the 161 first responders from the Province of Parma participating in the survey (note: SIV = Seasonal Influenza Vaccine; GP = General Practitioner; OPh = Occupational Physician; SD = Standard Deviation). In the survey, a “positive” vaccination status was defined as follows: two shots for measles, one booster shot against pertussis within the last 10 years, one shot for SIV for either the influenza season 2017 and/or 2018 (separately assessed). Regarding meningococcus, as several serotype-specific vaccines are available, any previous reported vaccination was considered appropriate.

	No./161, %	Average ± S.D.
Sex		
Male	103, 64.0%	
Female	58, 36.0%	
Age (years)		45.1 ± 14.1
≥50 y.o.	69, 42.9%	
Seniority (years)		10.8 ± 8.6
≥10 years	77, 47.8%	
Migration background	8, 5.0%	
Educational achievements		
0–8 years (primary school)	13, 8.1%	
9–13 years (secondary school/high school)	105, 65.2%	
14 or more (university or higher)	43, 26.7%	
Occupational background from healthcare settings	19, 11.8%	
Knowledge Status		
General Knowledge Score (%)		46.5% ± 32.4
Knowledge of the official recommendation from the National Immunization Prevention Plan 2017–2019 (%)		54.1% ± 11.8
Somehow favorable towards ...		
Seasonal Influenza Vaccine	89, 55.3%	
Pertussis Vaccine	132, 82.0%	
Measles Vaccine	141, 87.5%	
Meningococcal Vaccines	144, 89.4%	
SIV, perceived facilitators in subjects somehow favorable	No./89, %	
Protecting subjects who cannot be vaccinated	66, 74.2%	
Avoid spreading of seasonal influenza	67, 75.3%	
Avoid complications (respondent)	60, 67.4%	
Avoid natural infection (respondent)	41, 46.1%	
It was recommended by GP	5, 5.6%	
It was recommended by an OPh	5, 5.6%	
I bear some specific recommendations	13, 14.6%	
SIV, perceived barriers in subjects somehow not favorable	No./72, %	
Reputed as unnecessary (I’m otherwise immunized)	11, 15.3%	
Fear of injections	1, 1.4%	
Preference to other preventive measures	27, 37.5%	
Not enough trust in SIV	32, 44.4%	
SIV is useless	2, 2.8%	
Lifestyles are more efficient	2, 2.8%	
Fear of side effects	10, 13.9%	
Previously Vaccinated against ... (self-reported)		
Influenza, 2018	45, 28.0%	
Influenza, 2017	42, 26.1%	
Pertussis	56, 34.8%	
Measles	68, 42.2%	
Meningococcus	42, 26.1%	

**Table 2:** Assessment of general knowledge on immunizations, and awareness of specific recommendations (National Immunization Prevention Plan 2017–2019, PNPV 2017–2019) for healthcare workers among 161 first responders participating in the survey (Province of Parma, 2018). Notes: HAV = Hepatitis A Virus; HBV = Hepatitis B Virus; HPV = Human Papillomavirus; NVPP = National Vaccine Prevention Plan.

Statement	Correct Answer	No./161, %
The additives used in the vaccines are not dangerous to humans	TRUE	61, 37.9%
Multiple Sclerosis may be induced by the HBV vaccine	FALSE	69, 42.9%
Subacute sclerosing panencephalitis may be induced by the measles vaccine	FALSE	67, 41.6%
Autism is more frequent in subjects who have received the measles vaccine	FALSE	77, 47.8%
Diabetes mellitus may be triggered by vaccination shoots	FALSE	75, 46.6%
Vaccinations increase the occurrence of autoimmune diseases	FALSE	52, 32.3%
Vaccinations increase the risk of allergic disorders	FALSE	58, 36.0%
Vaccines are superfluous, as infectious diseases can always be treated with antibiotics	FALSE	96, 59.6%
Without massive vaccination programs, smallpox would still exist	TRUE	109, 67.7%
The efficacy of vaccines has been extensively proven	TRUE	103, 64.0%
Children would be more resistant to infections if they were not always treated against all diseases	FALSE	80, 49.7%
Many vaccinations are administered too early. As a result, the immune system has no possibility to fully develop by itself	FALSE	66, 41.0%
The immune system of children may be overwhelmed by a high number of vaccines	FALSE	61, 37.9%
Recommendations of Italian PNPV 2017–2019		
Diphtheria	NO	96, 59.6%
Tetanus	NO	25, 15.5%
Pertussis	YES	64, 39.8%
Poliomyelitis	NO	109, 67.7%
HAV	NO	56, 34.8%
HBV	YES	148, 91.9%
Influenza	YES	142, 88.2%
Pneumococcus	NO	76, 47.2%
H influenzae	NO	116, 72.0%
Measles	YES	98, 60.9%
Rubella	YES	83, 51.6%
Parotitis	YES	69, 42.9%
Varicella	YES	83, 51.6%
Meningitis	NO	55, 34.2%
HPV	NO	111, 68.9%
Tuberculosis (BCG)	NO	77, 47.8%

**Table 3:** Correlation between general knowledge score and risk perception score for natural infection and vaccination for the four assessed vaccine-preventable diseases. The analyses were performed via calculation using Spearman’s rank correlation coefficient.

	Risk Perception on…	
	Natural Infection	Vaccine
Seasonal Influenza	−0.081 (*p* = 0.310)	−0.239 (*p* = 0.002)
Pertussis	0.035 (*p* = 0.656)	−0.294 (*p* < 0.001)
Measles	0.151 (*p* = 0.057)	−0.278 (*p* < 0.001)
*N meningitidis* infections	0.161 (*p* = 0.042)	−0.260 (*p* = 0.001)

**Table 4:** Univariate analysis of the association between individual factors and having been vaccinated against seasonal influenza (SIV) 2018, pertussis (Pa), measles (MeV), meningitis (any vaccine, MEN). Note: HCW = healthcare worker.

Variables	SIV 2018			Pa			MeV			MEN		
	SIV pos. (No./45, %)	SIV neg. (No./116, %)	*p* Value	Pa pos. (No./56, %)	Pa neg. (No./105, %)	*p* Value	MeV pos. (No./68, %)	MeV neg. (No./92, %)	*p* Value	MEN pos. (No./45)	Men neg. (No./116)	*p* Value
Age ≥ 50 years	20, 44.4%	49, 42.2%	0.939	12, 21.4%	57, 54.3%	<0.001	13, 19.1%	56, 60.9%	<0.001	11, 26.2%	58, 48.7%	0.018
Male Sex	35, 77.8%	68, 58.6%	0.037	36, 64.3%	67, 63.8%	1.000	45, 66.2%	57, 62.0%	0.702	30, 71.4%	73, 61.3%	0.325
Migration background	1, 2.2%	7, 6.0%	0.552	7, 12.5%	1, 1.0%	0.005	7, 10.3%	1, 1.1%	0.023	2, 4.8%	6, 5.0%	1.000
Education ≥ University	10, 22.2%	33, 28.4%	0.547	21, 37.5%	22, 21.0%	0.038	25, 36.8%	18, 19.6%	0.025	7, 16.7%	36, 30.3%	0.132
Seniority ≥ 10 years	31, 68.9%	46, 39.7%	0.002	16, 28.6%	61, 58.1%	0.001	21, 30.9%	56, 60.9%	<0.001	20, 47.6%	57, 47.9%	1.000
Healthcare background	10, 22.2%	9, 7.8%	0.023	12, 21.4%	7, 6.7%	0.012	7, 10.3%	12, 13.0%	0.090	5, 11.9%	14, 11.8%	1.000
Knowledge of Official recommendations for HCW	25, 55.6%	49, 42.2%	0.179	29, 51.8%	45, 42.9%	0.359	33, 48.5%	40, 43.5%	0.636	21, 46.7%	53, 44.5%	0.667
General Knowledge Score > median	20, 44.4%	56, 48.3%	0.794	27, 48.2%	49, 46.7%	0.983	35, 51.5%	41, 44.6%	0.481	23, 54.8%	53, 44.5%	0.336
Risk Perception Score for Vaccine > median	18, 40.0%	41, 35.3%	0.713	20, 35.7%	24, 22.9%	0.119	21, 30.9%	27, 29.3%	0.972	14, 33.3%	43, 36.1%	0.890
Risk Perception Score for Natural infection > median	27, 60.0%	42, 36.2%	0.010	31, 55.4%	38, 36.2%	0.030	37, 54.4%	35, 38.0%	0.038	15, 35.7%	58, 48.7%	0.201
Somehow Favorable attitude	36, 80.0%	53, 45.7%	<0.001	48, 85.7%	84, 80.0%	0.494	60, 88.2%	80, 87.0%	1.000	39, 92.9%	105, 88.2%	0.585

**Table 5:** Multivariable analysis of the association between individual factors and having been vaccinated against seasonal influenza (SIV) 2018, pertussis (Pa), measles (MeV), meningitis (any vaccine, MEN). Multivariate odds ratios (aOR), with their respective 95% confidence intervals (95% CI) were calculated through binary regression analysis. The models included, as an outcome variable, being previously vaccinated, and as an effector variable, all demographic factors and individual factors in which univariate analyses were associated with a vaccination status with *p* < 0.05. Note: HCW = healthcare worker.

Variable	SIV 2018		Pa		MeV		MEN	
	aOR	95%CI	aOR	95%CI	aOR	95%CI	aOR	95%CI
Age ≥ 50 years	1.116	(0.471; 2.645)	0.169	(0.068; 0.420)	0.114	(0.047; 0.278)	0.316	(0.142; 0.704)
Male Sex	1.753	(0.689; 4.459)	0.968	(0.427; 2.261)	1.316	(0.579; 2.993)	1.482	(0.663; 3.312)
Migration background	0.820	(0.085; 7.881)	15.330	(1.418; 165.737)	6.958	(0.638; 75.839)	0.903	(0.163; 5.003)
Education ≥ University	0.537	(0.198; 1.454)	3.274	(1.291; 8.304)	2.693	(1.090; 6.651)	0.376	(0.147; 0.964)
Seniority ≥ 10 years	3.262	(1.346; 7.905)	0.198	(0.080; 0.489)	0.197	(0.085; 0.445)	-	-
Healthcare background	3.184	(0.908; 11.167)	10.898	(2.638; 45.017)	-	-	-	-
Risk Perception Score for Natural infection > median	3.374	(1.367; 8.332)	1.266	(0.547; 2.932)	1.285	(0.502; 3.285)	-	-
Somehow Favorable attitude	8.404	(3.070; 23.009)	-	-	-	-	-	-

## Data Availability

The data presented in this study are available on request from the corresponding author.

## Supplementary Materials

The following are available online at https://www.mdpi.com/article/10.3390/vaccines10091492/s1, Table S1. STROBE Statement Checklist; Supplementary material File S2. Author’s translation of the Informed Consent.
